# The Anti-Inflammatory Activity of Boron Derivatives in Rodents

**DOI:** 10.1155/MBD.1995.1

**Published:** 1995

**Authors:** Iris H. Hall, Bruce S. Burnham, Shang Y. Chen, Anup Sood, Bernard F. Spielvogel, Karen W. Morse

**Affiliations:** 1 Division of Medicinal Chemistry and Natural Products School of Pharmacy University of North Carolina Chapel Hill North Carolina 27599-7360 USA; 2 Boron Biologicals, Inc. 620 Hutton street Raleigh North Carolina 27606 USA; 3 Department of Chemistry and Biochemistry Utah State University Logan Utah 84322 USA

## Abstract

Acyclic amine-carboxyboranes were effective anti-inflammatory agents in mice at 8 mg/kg x 2. These amine-carboxyboranes were more effective than the standard indomethacin at 8 mg/kg x 2, pentoxifylline at 50 mg/kg x 2, and phenylbutazone at 50 mg/kg x 2. The heterocyclic amine derivatives as well as amine-carbamoylboranes, carboalkoxyboranes, and cyanoboranes were generally less active. However, selected aminomethyl-phosphonate-N-cyanoboranes demonstrated greater than 60% reduction of induced inflammation. The boron compounds were also active in the rat induced edema, chronic arthritis, and pleurisy screens, demonstrating activity similar to the standard indomethacin. The compounds were effecive in reducing local pain and decreased the tail flick reflex to pain. The derivatives which demonstrated good anti-inflammatory activity were effective inhibitors of hydrolytic lysosomal, and proteolytic enzyme activities with IC_50_ 50 values equal to ^-6^M in mouse macrophages, human leukocytes, and Be Sal osteofibrolytic cells. In these same cell lines, the agents blocked prostaglandin cyclooxygenase activity with IC_50_ values of ^-6^M. In mouse macrophage and human leukocytes, 5′ lipoxygenase activity was also inhibited by the boron derivatives with IC_50_ values of 10^-6^M. These IC_50_ values for inhibition of these enzyme activities are consistent with published values of known anti-inflammatory agents which target these enzymes.


### THE ANTI-INFLAMMATORY ACTIVITY OF BORON DERIVATIVES IN RODENTS

Iris H. Hall[1], Bruce S. Burnham[1], Shang Y. Chen[1], Anup Sood[2], Bernard F. Spielvogel[2] and Karen W. Morse[3]

[1] Division of Medicinal Chemistry and Natural Products, School of Pharmacy, University of North Carolina, Chapel Hill, North Carolina 27599-7360, USA
[2] Boron Biologicals, Inc., 620 Hutton Street,Raleigh, North Carolina 27606, USA
[3] Department of Chemistry and Biochemistry, Utah State University, Logan, Utah 84322, USA

### ABSTRACT

Acyclic amine-carboxyboranes were effective anti-inflammatory agents in mice at 8 mg/kg x 2. These amine-carboxyboranes were more effective than the standard indomethacin at 8 mg/kg x 2, pentoxifylline at 50 mg/kg x 2, and phenylbutazone at 50 mg/kg x 2. The heterocyclic amine derivatives as well as amine-carbamoylboranes, carboalkoxyboranes, and cyanoboranes were generally less active. However, selected aminomethyl-phosphonate-N-cyanoboranes demonstrated greater than 60% reduction of induced inflammation. The boron compounds were also active in the rat induced edema, chronic arthritis, and pleurisy screens, demonstrating activity similar to the standard indomethacin. The compounds were effective in reducing local pain and decreased the tail flick reflex to pain. The derivatives which demonstrated good anti-inflammatory activity were effective inhibitors of hydrolytic lysosomal, and proteolytic enzyme activities with $IC_{50}$ values equal to $10^{-6}$M in mouse macrophages, human leukocytes, and Be Sal osteofibrolytic cells. In these same cell lines, the agents blocked prostaglandin cyclooxygenase activity with $IC_{50}$ values of $10^{-6}$M. In mouse macrophage and human leukocytes, 5' lipoxygenase activity was also inhibited by the boron derivatives with $IC_{50}$ values of $10^{-6}$M. These $IC_{50}$ values for inhibition of these enzyme activities are consistent with published values of known anti-inflammatory agents which target these enzymes.

### INTRODUCTION

Amine-cyanoboranes and amine-carboxyboranes have previously been shown to inhibit induced inflammation in rodents at 10 mg/kg[1]. These studies were expanded to include di- and tripeptides containing boron[2], tricyclohexyl- and triphenylphosphine- boranes, carboxyboranes and cyanoboranes[3], and boronated 2'-deoxynucleosides[4] which demonstrated anti-inflammatory activity in mice. These boron compounds were potent inhibitors of lysosomal hydrolytic enzyme activities and their release from $CF_1$-mouse hepatic tissue and human PMNs[1]. The current study extends this investigation to other boron derivatives and additional inflammatory disease models other than induced edema. Selective derivatives of individual chemical classes of boron compounds are examined for their mode of action using isolated enzymes and tissue cultured cells to determine their effects on hydrolytic and proteolytic enzymes and regulatory enzymes of the prostaglandin and leukotriene synthetic pathways.

### METHODS AND MATERIALS

Source of Materials
All of the boron derivatives have previously been synthesized and the literature references[5-24] for each compound are listed in Table 1. The chemical and physical characteristics were identical to those reported in the literature. Test compounds (1-94) were homogenized in 0.05% tween 80-distilled/$H_2O$.

Anti-inflammatory Activity. $CF_1$ male mice (~25g) were administered test drugs (1-94) at 8 mg/kg/day in 0.05% Tween 80/$H_2O$ intraperitoneally at 3 h and again at 30 min prior to the injection of 0.2 ml of 10% carrageenan in 0.9% saline into the plantar surface of the right hind foot. Saline was injected into the left hind foot which served as the standard base line. After 3 h, both feet were excised at the tibiotarsal (ankle) joint according to the modified method of Winter[25,26], resulting in an 84 mg increase in the paw weight of the control mice. Selected compounds 2, 4, 5, 6, 8, 9, 11, 12, 15, 16, 23, 33, 34, 36, 46, 48, 74, 76, and 78 were tested in Sprague Dawley male rats (~350 g) at 8 mg/kg x 2, I.P. The average paw weight of the control rats was increased 1.732 g by the inflammation agent.

Local Analgesic Screen. Male $CF_1$ mice (~25 g) were administered selected test drugs (1-89) at 8 mg/kg, I.P. 20 min prior to the administration of 0.5 ml of 0.6% acetic acid, I.P. After 5 min, the number of stretches, characterized by repeated contractions of the abdominal musculature, accompanied by the extension of the hind limbs, were counted for the next 10 min[25]. The control mice afforded 61 stretch reflexes per 10 min.

Hot Plate Tail Flick Screen. $CF_1$ male mice (~28 g) were administered agents at 8 mg/kg, I.P. 15 min prior to placement on a hot plate maintained at 100°C. The time elapse for the tail to be raised was determined using a digital read-out connected to the hot plate[27]. Tail flick responses of mice injected with morphine was used as a standard.

Anti-pleurisy Screen. Sprague Dawley male rats (~280 g) were administered test drugs 2, 8, 9, 12, 15, 16, 20, 34, or 76 at 8 mg/kg I.P., 1 h before and 3 h after injecting into the pleural cavity 0.05 ml of a 0.316% Evan's blue and carrageenan[28]. Six hours later, the rats were sacrificed and the fluid collected from the pleural cavity. Control rats produced an average of 2.15 ml of fluid.

Chronic Adjuvant Arthritic Screen. Male Sprague Dawley rats (~180 g) were injected at the base of the tail with 0.2 ml of solution containing 100 mg of dried M. butyricum and 40 mg digitonin in 20 ml of light mineral oil[29]. Test drugs 2, 7, 8, 12, 20, 34, or 78 were administered on Day 3 to Day 21 at 8 mg/kg/day, I.P. The following day, the rats were sacrificed and the feet were excised and weighed. Control rats averaged a 0.750 g net weight increase.

Cell Maintenance of Cultured Cells. Mouse macrophages J774A were maintained in Dulbecco's modified Eagle medium (DMEM) + 15% FCS + P/S. RMPI 1788 human leukocytes were maintained in RMPI 1640 + 10% FCS + P/S. Be Sal human osteo-fibrolytic cells were maintained in RMPI + 10% FCS + P/S. Rat osteogenic sarcoma cells UMR-106 were maintained in DMEM + 10% FCS + P/S.

Lysosomal Hydrolytic Enzymatic Activities. Acid phosphatase, aryl sulphatase, cathepsin, and trypsin proteolytic activities were measured utilizing human Be Sal osteo-fibrolytic osteoporosis cells, leukocytes, or mouse macrophages J774 (1 x $10^6$ cells). Drugs were incubated from $10^{-8}$ to $10^{-3}$ M for 30 min at 37°C. Acid phosphatase activity was determined using 0.1 M β-glycerolphosphate in 0.1 M acetate buffer at pH 5.0[32]. The reaction was stopped with 10% TCA and centrifuged at 3000 g x 6 min. The supernatant inorganic phosphate was determined by the spectrophotometric method of Chen et. al.[30]. The net inorganic phosphate released in 30 min was corrected by subtracting the blank test. Aryl sulphatase activity was measured using 0.72 mmole of p-nitrocatechol sulfate as a substrate in 0.2 M acetate buffer, pH 5.0 for 30 min at 37° C[31]. The reaction was terminated with 4N NaOH. The formation of 4-nitrocatechol in the supernatant was measured spectrophotometrically at 510 nm and corrected for blank values. Cathepsin activity was determined using 2% azocasien as the substrate in 0.1 M acetate buffer, pH 5.0 for 30 min at 37°C[31]. The reaction was terminated with 10% TCA and centrifuged. The supernatant was assayed for acid-soluble peptide fragments at 366 nm and corrected for blank values.

Proteolytic Enzyme Activities. Trypsin proteolytic activity was determined by the method of Schleuning and Fritz[33] using 2.0 ml of 0.1 M Tris buffer, pH 8.0 and 6 mM of N-benzoyl-L-arginine ethyl ester (BAEE) substrate. The hydrolysis of BAEE over a 30 min period was determined at 253 nm and blank values were subtracted[33]. Elastase activity was determined by the method of Kleinerman et al.[34] using 2.9 ml of 0.2 M Tris-HCl buffer, pH 8.0, 2 units (200 µl) of porcine pancreatic elastase (Sigma, Type III) and 20 µl N-succinyl-L-alanyl-L-alanine-p-nitroanilide (Sigma, 100 mg in 5 ml of methyl-2-pyrrolidone). The cleaved product p-nitroanilide was determined at 410 nm for a 3 min period. Collagenase activity was determined at 410 nm for a 3 min period. Collagenase activity was determined by the method of Hu et al.[35] using 1 ml of 50 mM Tris + 5 mM $CaCl_2$ buffer, pH 7.6, 10 µg $^3$H-collagen N-(propionate 2,3-$^3$H)propionylated (0.51 mCi/mg) and 10 µg collagenase (Sigma, Type III Clostridium histolyticum)[36] incubated for 24 h at 37°C. The reaction was stopped with 1 ml 50 mM EDTA. The tubes were then centrifuged at 10,000 g for 15 min. The supernatant was counted (Packard Tricarb liquid scintillation counter). Background radioactivity values were subtracted from each sample and corrected for quenching.

Prostaglandin Synthetase Activity. The incubation medium of Tomlinson et al.[37] was used to determine prostaglandin formation et al. from $^3$H(N)-arachidonic acid (100 Ci/mmole) and 10 mg of purified commercial prostaglandin synthetase from bovine seminal vesicles (Miles Research Products) or $10^6$ J774 macrophage cells. After 1 h, the reaction was terminated with 2N HCl and the mixture was extracted with ether and evaporated. The residue was dissolved in ethyl acetate and spotted on silica gel TLC plates which were eluted with chloroform, methanol, water, and acetic acid (90:8:1:0.8). The plates were developed in iodine vapor and the area appropriate to prostaglandin standards was scraped and counted[38], i.e. arachidonic acid, $PgA_2$, $PgE_2$, and $PgF_{2}\alpha$. The dpm in each area was calculated as percent of the total dpm on the tlc plate.

5'Lipoxygenase Activity. Mouse macrophages or human leukocytes (5 x $10^6$ cells) were maintained as described above and then incubated at $10^{-4}$ to $10^{-8}$ M. The cells were harvested by centrifugation and incubated in a phosphate buffer, pH 7.2, + 0.6 mM $CaCl_2$ + 1.0 mM $MgCl_2$, the calcium ionophore A 23187, and $^3$H-arachidonic acid. After 20 min incubation at 37°C, EtOAc:$CH_2Cl_2$ (2:3) supplemented with 12 mg arachidonic acid was added. The organic phase was extracted and dried. The residue was taken up in ethyl acetate and 100 µL was plated on silica gel TLC plates eluted with methylene chloride:methanol:acetic acid: water (90:8:1:0.8). The plates were scaped in the area that corresponded to 5-HETE and counted[39-40]. The $IC_{50}$ values were calculated for the compounds.

In Table 1, the anti-inflammatory activity is represented as percentage of control and the standard deviation (X ± S.D.). N equals the number of animals used in each test group. $IC_{50}$ values were calculated as the dose (log) which afforded 50% inhibition of the enzyme activity ± S.D. The P values were determined using the Student's "t" test and analysis of variance.

### RESULTS

In the mouse edema screen, compounds. 15, 16, and 19, the methyl and ethyl ester derivatives of amine-carboxyboranes 13-20 were less active than the simple acids (Table 1). The amides 21-31, except compound 23, were also less active than the acids. The ammonia di- and trimethylamine cyanoboranes 32-34 demonstrated good activity as did the $C_{18}H_{37}$ derivative 36 and the dicyano derivative 40. Nevertheless, the remaining cyanoboranes demonstrated minimum activity. The phosphonoacetate analogs and aminoalkylphosphonate derivatives demonstrated good activity where compounds 54, 62, 64, 66, 67, 68, 69, 71, 73, 74, 75, and 76 caused at least 40% reduction of induced edema in mice at 8 mg/kg x 2. The heterocyclic amine boranes derivatives 78-94 were not as active as other boron containing derivatives. Compound 78 caused 45% reduction of induced edema. Compounds 83, 84, 87, and 91 resulted in at least 30% reduction of inflammation in mice.

Compounds 4, 5, 6, 8, 15, and 23 demonstrated at least 40% reduction in induced edema in rats at 8 mg/kg x 2 (Table 2). Chronic arthritis in rats was reduced significantly by 7, 20, and 34 with greater than 75% reduction of pannus at 8 mg/kg/day. Compounds 2, 8, 12, and 78 were significantly active in this screen. Pleurisy edema in rats was reduced significantly by compounds 2, 8, 9, 12, 16, 20, 34, and 76. Selective agents were able to block local pain induced by acetic acid in mice. Compounds 1, 4, 5, 8, 9, 12, 15, 16, 33, 34, 35, and 78 reduced the reflex action by 80%. Compounds 2, 7, 20, 23, 27, 36, 46, 48, 74, 75, 76, and 77 caused at least 45% reduction of the reflex action. The tail flick assay time was increased by compounds 2, 3, 5, 8, 9, 12, 15, 16, 46, 75, and 76; however, only compounds 9, 75, and 76 were as effective as morphine in blocking central pain.

Lysosomal enzyme activity, e.g. acid phosphatase, cathepsin, and aryl sulfatase activities (Table 3) were inhibited by the boron derivatives. Acid phosphatase activity in mouse macrophages demonstrated $IC_{50}$ values between 0.90-7.72 x $10^{-6}$M for the boron derivatives tested, for acid cathepsin activity values between 0.76-9.45 x $10^{-6}$M, and for aryl sulfatase activity values between 1.37-9.71 $10^{-6}$M, except for compound 14 (17.6 x $10^{-6}$M). Human Be Sal cells showed $IC_{50}$ values for acid phosphatase activity between 1.10-18.3 x $10^{-6}$M, for acid cathepsin activity values between 0.85-6.17 x $10^{-6}$M, and for aryl sulfatase activity values between 6.07-10.00 x $10^{-6}$M. For human leukocytes acid phosphatase activity, the $IC_{50}$ values (Table 4) were between 8.57-10.49 x $10^{-6}$M, and acid cathepsin activity values between 5.38-7.71 x $10^{-6}$M. Proteolytic enzyme activities were also reduced by the boron derivatives. Macrophage trypsin activity (Table 5) was inhibited with $IC_{50}$ values between 1.26-4.34 x $10^{-6}$M, macrophage elastase activity was inhibited with $IC_{50}$ values ranging from 0.22-9.01 x $10^{-6}$M. Be Sal trypsin activity demonstrated inhibition with $IC_{50}$ values between 3.35-7.40 x $10^{-6}$M (Table 4) whereas elastase activity was inhibited with $IC_{50}$ values in the range of 0.58-3.86 x $10^{-6}$M. Macrophage trypsin and elastase activities were inhibited by the compounds with $IC_{50}$ values ~ $10^{-6}$M. Commercial collagenase I activity was inhibited with $IC_{50}$ values between 615-812 x $10^{-6}$M (Table 5) and type II activity with $IC_{50}$ values between 89-124 x $10^{-6}$M. Human bone osteogenic sarcoma cells (Table 6) trypsin activity was inhibited with $IC_{50}$ values of 5.67-10.51 x $10^{-6}$M, elastase activity values of 8.09-10.34 x $10^{-6}$M, and acid cathepsin activity values between 4.67-6.62 x $10^{-6}$M. Beef seminal vesicle prostaglandin cyclooxygenase activity (Table 7) was inhibited by the boron derivatives with $IC_{50}$ values ranging from 0.71-7.60 x $10^{-6}$M. Macrophage prostaglandin cyclooxygenase activity $IC_{50}$ values were between 1.47-7.72 x $10^{-6}$M, and Be Sal cells showed $IC_{50}$ values between 1.09-8.04 x $10^{-6}$M. Human leukocyte 5'-lipoxygenase activity was inhibited by the boron derivatives with $IC_{50}$ values between 3.47-6.43 x $10^{-6}$M.

**Table 1. The Anti-inflammatory Activity of Boron Derivatives in CF-1 Mice at 8 mg/kg x 2, I.P.**

| N = 6 | Compound | Anti-inflam-matory % Control | Synthetic Reference | | Compound | Anti-inflam-matory % Control | Synthetic Reference |
|---|---|---|---|---|---|---|---|
| 1. | $(CH_3)_3CNH_2BH_3$ | 41 | Alfa Products | 28. | $(CH_3)_3NBH_2C(O)NH(n\text{-}(CH_2)_7CH$ | 86 | 6 |
| 2. | $CH_3NH_2BH_2COOH$ | 54 | 5 | 29. | $(CH_3)_3NBH_2C(O)NHC_6H_5$ | 72 | 6 |
| 3. | $(CH_3)_2NHBH_2COOH$ | 59 | 5 | 30. | $[CH_2N(CH_3)_2BH_2C(O)NHC_2H_5]_2$ | 65 | 7 |
| 4. | $(CH_3)_3NBH_2COOH$ | 59 | 1,5 | 31. | $(CH_3)_3NBH(CH_3)C(O)NHC_2H_5$ | 83 | 14 |
| 5. | $CH_3CH_2NH_2BH_2COOH$ | 59 | 6 | 32 | $NH_3BH_2CN$ | 51 | 15 |
| 6. | $(n\text{-}C_3H_7)_2NHBH_2COOH$ | 81 | 6 | 33. | $(CH_3)_2NHBH_2CN$ | 49 | 1 |
| 7. | $\text{-}[CH_2N(CH_3)_2BH_2COOH]_2$ | 58 | 7 | 34. | $(CH_3)_3NBH_2CN$ | 42 | 1,3 |
| 8. | $C_{16}H_{33}N(CH_3)_2BH_2COOH$ | 40 | 8 | 35. | $(CH_3)_2(C_{16}H_{33})NBH_2C$ | 75 | 8 |
| 9. | $C_{18}H_{37}N(CH_3)_2BH_2COOH$ | 58 | 8 | 36. | $(CH_3)_2(C_{18}H_{37})NBH_2CN$ | 59 | 8 |
| 10. | $H_2NNH_2BH_2COOH$ | 82 | 6 | 37. | $(CH_3)_2NHBH(CH(CH_3)_2)CN$ | 78 | 16 |
| 11. | $C_5H_5NBH_2COOH$ | 40 | 9 | 38. | $(CH_3)_2NHB(CH(CH_3)_2)_2CN$ | 79 | 16 |
| 12. | $(C_6H_5)_3PBH_2COOH$ | 40 | 10 | 39. | $CH_3)_3NBH(CH_3)CN$ | 94 | 14 |
| 13. | $H_3NBH_2COOCH_3$ | 66 | 11 | 40. | $(CH_3)_3NB(CN)_2CH_3$ | 59 | 14 |
| 14. | $CH_3NH_2BH_2COOCH_3$ | 79 | 11 | 41. | $\text{-}[CH_2NH_2BH_2CN]_2$ | 87 | 7 |
| 15. | $(CH_3)_2NHBH_2COOCH_3$ | 58 | 7 | 42. | $\text{-}[CH_2N(CH_3)_2BH_2CN]_2$ | 95 | 7 |
| 16. | $(CH_3)_3NBH_2COOCH_3$ | 58 | 11 | 43. | $NCCH_2CH_2N(CH_3)_2BH_2CN$ | 74 | 17 |
| 17. | $n\text{-}CH_3CH_2CH_2CH_2NH_2BH_2COOCH_3$ | 71 | 6 | 44. | $(CH_3)_3NBH_2CH_2CN$ | 78 | 18 |
| 18. | $C_{16}H_{33}N(CH_3)_2BH_2COOCH_3$ | 71 | 8 | 45. | $(CH_3)_3NBH_2C(CH_3)_2CN$ | 64 | 18 |
| 19. | $C_{16}H_{33}N(CH_3)_2BH_2COOCH_2CH_3$ | 53 | 8 | 46. | $(CH_3)_3NBH_2CH(C_6H_5)CN$ | 80 | 18 |
| 20. | $(CH_3)_2NHBH_2COOCH_2CH_3$ | 76 | 11 | 47. | $C_5H_5NBH_2C(CH_3)_2CN$ | 64 | 18 |
| 21. | $(BH_2C(O)NHCH_2CH_3)_2$ | 104 | 12 | 48. | $(C_6H_5)_3PBH_2CN$ | 73 | 9,19 |
| 22. | $H_3NBH_2C(O)NHC_2H_5$ | 76 | 13 | 49. | $(C_2H_5O)_3PBH_2CN$ | 94 | 20 |
| 23. | $(CH_3)NH_2BH_2C(O)NHC_2H_5$ | 51 | 13 | 50. | $(C_2H_5O)_3PBH_2C(O)NHCH_2CH_3$ | 64 | 20 |
| 24. | $(CH_3)_2NHBH_2C(O)NHC_2H_5$ | 84 | 13 | 51. | $(C_2H_5O)_3PBH_2COOH$ | 73 | 20 |
| 25. | $(CH_3)_3NBH_2C(O)NHC_2H_5$ | 61 | 13 | 52. | $(C_2H_5O)_3PBH_2COOCH_3$ | 66 | 20 |
| 26. | $(CH_3)_3NBH_2C(O)NH(n\text{-}CH_2CH_2CH_3)$ | 61 | 6 | 53. | $(C_2H_5O)_2(NaO)PBH_2CN$ | 66 | 20 |
| 27. | $(CH_3)_3NBH_2C(O)NH(n\text{-}(CH_2)_3CH_3)$ | 86 | 6 | 54. | $(C_2H_5O)_2(NaO)PBH_2CONHC_2H_5$ | 56 | 20 |

Table 1. (cont.)

| N = 6 | Compound | Anti-inflam-matory % Control | Synthetic Reference | | Compound | Anti-inflam-matory % Control | Synthetic Reference |
|---|---|---|---|---|---|---|---|
| 55. | $(C_2H_5O)_2(NaO)PBH_2COOCH_3$ | 66 | 20 | 77. | $(CH_3)_3NBH_2C(O)NHOH.HCl$ | 78 | 23 |
| 56. | $(C_4H_9)_4N{+}(C_2H_5O)_2(^-O)PBH_2CN$ | 89 | 20 | 78. | Morpholine-borane | 55 | 24 |
| 57. | $(C_4H_9)_4N^+(C_2H_5O)_2(^-O)PBH_2$ $ONHC_2H_5$ | 97 | 20 | 79. | Piperidine-cyanoborane | 77 | 24 |
| 58 | $(C_4H_9)_4N^+(C_2H_5O)_2(^-O)PBH_2COOCH_3$ | 67 | 20 | 80. | Piperazine-cyanoborane | 83 | 24 |
| 59. | $(C_2H_5O)(HO)PBH_2CN$ | 78 | 20 | 81. | Piperidine-carboxyborane | 84 | 24 |
| 60. | $Na{+}H^+(C_2H_5O_2)(^-O)PBH_2COO^-$ | 20 | | 82. | Piperazine-carboxyborane | 79 | 24 |
| 61. | $((C_4H_9)_3NH^+)_2(C_2H_5O)(^-O)_2PBH_2CN$ | 89 | 20 | 83. | Morpholine-carboxyborane | 66 | 24 |
| 62. | $(CH_3O)_2P(O)CH_2N(C_2H_5)_2BH_2CN$ | 38 | 21 | 84. | N-Methylmorpholine-carboxyborane | 67 | 24 |
| 63. | $(C_2H_5O)_2P(O)CH_2N(C_2H_5)_2BH_2CN$ | 68 | 218 | 85. | N-Methylmorpholine-cyanoborane | 91 | 24 |
| 64. | $(i\text{-}C_3H_7O)_2P(O)CH_2N(C_2H_5)_2BH_2CN$ | 36 | 21 | 86. | 4-Phenylpiperidine-carboxyborane | 80 | 24 |
| 65. | $(C_6H_5O)_2P(O)CH_2NH_2BH_2CN$ | 67 | 21 | 87. | 4-Phenylpiperidine-carbomethoxyborane | 67 | 24 |
| 66. | $(C_2H_5O)_2P(O)CH_2N(CH_3)_2BH_2CN$ | 48 | 21 | 88. | 4-Methylpiperidine-carboxyborane | 80 | 24 |
| 67. | $(i\text{-}C_3H_7O)_2P(O)CH_2NH(CH_3)BH_2CN$ | 48 | 21 | 89. | Imidazole-cyanoborane | 99 | 24 |
| 68. | $(CH_3CH_2O)_2P(O)CH_2N(CH_3)_2BH_2COOCH_2CH_3$ | 54 | 22 | 90. | $N^1$-Methylimidazole-$N^3$-cyanoborane | 71 | 24 |
| 69. | $(CH_2CH_2O)_2P(O)CH_2NH(CH_3)BH_2CONHCH_2CH_3$ | 42 | 22 | 91. | 2-Methylimidazole-$N^3$-cyanoborane | 62 | 24 |
| 70. | $(CH_3CH_2O)_2P(O)CH_2N(CH_3)_2BH_2COOH$ | 67 | 22 | 92. | (Trimethylamine-boryl)carbonyl)imidazole | 86 | 24 |
| 71. | $(i\text{-}Pro)_2P(O)CH_2NHCH_3BH_2COOH$ | 31 | 6 | 93. | $N^1$-Methylimidazole-$N^3$-carbomethoxyborane | 76 | 24 |
| 72. | $(i\text{-}Pro)_2P(O)CH_2NHCH_3BH_2CH_2CH_3$ | 65 | 6 | 94. | $N^1$-Methylimidazole-$N^3$-(N'-ethyl)carbamoylbor | 71 | 24 |
| 73. | $(i\text{-}Pro)_2P(O)CH_2NHCH_3BH_2CONHCH_2CH_3$ | 58 | 6 | | Indomethacin 8 mg/kg x 2 | 74 | |
| 74. | $(CH_3)_3NBH_2C{=}NCH_2CH_3BF_4^-$ | 48 | 5 | | 10 mg/kg x 2 | 23 | |
| 75. | $CH_3NH_2BH_2C(O)NHOH.HB(C_6H_5)_4$ | 44 | 23 | | Phenybutazone 50 mg/kg x 2 | 53 | |
| 76. | $(CH_3)_3NBH_2C(O)NHOH.HB(C_6H_5)_4$ | 56 | 23 | | Pentoxifylline 50 mg/kg x 2 | 70 | |

**Table 2. The in vivo Antiinflammatory and Analgesic Activities of Boron Derivativesin Rats and Mice at 8 mg/kg (% of control)**

| | Rat | | | Mice | |
|---|---|---|---|---|---|
| Compound | Inflammatory | Chronic Arthritis | Pleurisy | Writhing Reflex | Tail Flick |
| 1 | --- | --- | --- | 19 | --- |
| 2 | 70 | 53 | 61 | 31 | 147 |
| 3 | --- | --- | --- | --- | 156 |
| 4 | 55 | -- | -- | 2 | --- |
| 5 | 59 | --- | --- | 1 | 196 |
| 6 | 55 | --- | --- | 19 | --- |
| 7 | --- | 24 | --- | 55 | --- |
| 8 | 59 | 40 | 19 | 16 | 167 |
| 9 | 72 | --- | 37 | 14 | 231 |
| 11 | 77 | --- | --- | --- | --- |
| 12 | 61 | 49 | 28 | 19 | 197 |
| 15 | 59 | -- | 86 | 12 | 147 |
| 16 | 71 | --- | 45 | 2 | 180 |
| 20 | --- | 13 | 61 | 36 | --- |
| 23 | 41 | --- | --- | 27 | 81 |
| 25 | --- | --- | --- | 89 | --- |
| 30 | --- | --- | --- | 34 | --- |
| 33 | 61 | --- | --- | 15 | --- |
| 34 | 65 | 4 | 65 | 18 | --- |
| 35 | --- | --- | --- | 16 | --- |
| 36 | 74 | --- | --- | 61 | --- |
| 46 | --- | --- | --- | 46 | 191 |
| 48 | 78 | --- | --- | 50 | 134 |
| 74 | 81 | --- | --- | 40 | --- |
| 75 | --- | --- | --- | 34 | 279 |
| 76 | 60 | --- | 50 | 45 | 258 |
| 77 | --- | --- | --- | 41 | 139 |
| 78 | 83 | 33 | --- | 15 | --- |
| 87 | --- | --- | --- | 89 | --- |
| Indomethacin | 78 | 55 | 12 | 43 | --- |
| Morphine | | | | | 213 |
| Phenylbutazone | 47 | | | | |

### Discussion

The amine-carboxyboranes and related derivatives were not all active as anti-inflammatory agents in rodents at 8 mg/kg x 2. Nevertheless, selected compounds were equally active as the clinically used standards indomethacin, phenylbutazone, and pentoxifylline which afforded 26, 47, and 30% inhibition, respectively of induced inflammation at their therapeutic dose. A number of the amine-carboxyboranes and cyanoborane derivatives were more potent in reducing edema at 8 mg/kg x 2. The mode of actions of the active compounds appears to be similar to those previously reported for cyanoboranes[1] in that these agents effectively reduced the activities of lysosomal and proteolytic enzymes in a number of cultured cells. Particularly important is their inhibition on these processes in macrophages, leukocytes, and Be Sal osteo-fibrolytic cells. Studies have recently shown that the carboxyborane derivatives effectively inhibit the release of chemical mediators, e.g. tumor necrosis factor-alpha, Il-1 and Il-2 from macrophages and further, these same agents reduce the movement of PMNs and macrophages to sites of inflammation[41]. These chemical mediators, when released from inflammation cells, interact with high affinity receptors of target cells causing release of hydrolytic and proteolytic enzymes which cause local destruction of the tissue. Released locally also are prostaglandins and leukotrienes which afford additional inflammation, edema, pain, and destruction of tissue. Apparently, the boron derivatives are potent inhibitors of both prostaglandin cycooxygenase and 5'-lipoxygenase activities of human leukocytes and mouse macrophages with $IC_{50}$ values which are typical of anti-inflammatory agents, i.e. $10^{-6}$M. The fact that these agents are dual inhibitors is most significant since all agents are not able to achieve this function. The inhibition of 5'-lipoxygenase activity by the amine-carboxyboranes is potent achieving an order of magnitude greater inhibition than most dual inhibitors under investigation[41,42]. The amine-carboxyboranes, like most anti-inflammatory agents, function by multiple modes of action to reduce inflammation and pain.

**Table 3. $IC_{50}$ Values X $10^{-6}$M For Inhibition of Lysosomal Enzymes**

| | Mouse Macrophage | | | Human Be Sal Osteofibrolytic | | |
|---|---|---|---|---|---|---|
| Compound # | Acid Phosphatase | Cathepsin | Aryl Sulfatase | Acid Phosphatase | Cathepsin | Aryl Sulfatase |
| 1 | -- | 8.08 | 4.62 | 10.20 | 4.28 | 9.14 |
| 2 | 0.90 | 9.45 | 8.30 | | 1.77 | 8.86 |
| 3 | 4.75 | 6.39 | | | | |
| 7 | 3.32 | 8.37 | | | | |
| 8 | 0.94 | 7.18 | 9.71 | 2.06 | 1.37 | 6.07 |
| 9 | 5.34 | 5.09 | | | | |
| 11 | | | | 18.30 | 5.44 | 8.16 |
| 12 | 1.08 | 5.52 | 8.58 | 15.30 | 4.28 | 9.33 |
| 15 | 1.04 | 6.71 | 7.41 | 1.10 | | 8.59 |
| 16 | 3.55 | 8.28 | 17.60 | | 0.73 | |
| 20 | 1.12 | 3.94 | 9.27 | | 1.28 | 7.62 |
| 23 | 1.12 | 3.95 | 9.27 | 8.60 | 1.93 | 7.63 |
| 33 | 1.13 | 5.54 | 9.02 | 8.70 | 0.85 | 7.91 |
| 34 | 1.05 | 6.34 | 9.29 | 15.90 | 1.34 | 8.87 |
| 35 | 0.33 | 4.97 | 7.16 | 2.01 | 10.00 | |
| 36 | 2.68 | 3.69 | | | | |
| 40 | 1.10 | 6.89 | 8.48 | | | |
| 46 | 7.72 | 2.57 | 3.40 | | | |
| 57 | 4.43 | 6.49 | | | | |
| 59 | 2.04 | 6.55 | | | | |
| 74 | 1.31 | 9.13 | 6.03 | | 6.17 | 8.80 |
| 75 | 6.61 | 4.58 | 1.50 | | | |
| 76 | 3.96 | 1.69 | 1.37 | | | |
| 77 | 6.59 | 5.13 | 1.40 | | | |
| 78 | 3.18 | 0.76 | | 10.30 | 4.40 | 8.99 |
| 83 | 0.16 | 1.76 | 7.59 | | 2.93 | 8.99 |
| 87 | 2.68 | 3.99 | | | | |
| 95 | 3.24 | 9.24 | | | | |

**Table 4. $IC_{50}$ Values X $10^{-6}$ M For Inhibition of Human Enzyme Activities**

| | Be Sal Osteofibrolytic | | | Leukocytes | |
|---|---|---|---|---|---|
| Compound # | Trypsin | Elastase | Acid Phosphatase | Cathpsin | Trypsin |
| 1 | 7.00 | 3.15 | | | |
| 2 | 7.04 | 1.39 | --- | 7.03 | 8.50 |
| 8 | 7.40 | 3.86 | --- | 6.46 | |
| 12 | 5.70 | 1.96 | 7.25 | 11.00 | |
| 15 | 6.14 | 1.31 | | | |
| 16 | | | 9.94 | 5.97 | 6.72 |
| 20 | 4.36 | 1.97 | | | |
| 23 | 4.36 | 0.98 | 10.80 | 9.77 | |
| 33 | 6.43 | 3.36 | 10.49 | 5.38 | 11.31 |
| 34 | 3.98 | 3.29 | 10.03 | 7.71 | 9.95 |
| 35 | 5.87 | 1.65 | | | |
| 36 | | | 8.57 | | 7.25 |
| 40 | 3.35 | 1.21 | 6.52 | 10.62 | |
| 74 | 4.47 | 2.12 | 9.69 | | 9.68 |
| 76 | 3.35 | 3.60 | | | |
| 78 | 7.88 | 1.84 | 8.58 | 7.28 | 7.40 |
| 83 | 7.88 | 0.575 | | | |

**Table 5. Proteolytic Activity $IC_{50}$ Values x $10^{-6}$M for Boron Derivatives**

| | Mouse Macrophage | | Clostridium histolyticum | |
|---|---|---|---|---|
| Compound # | Trypsin | Elastase | Collagenase I | Collagenase II |
| 1 | 2.09 | 0.22 | --- | 101 |
| 2 | 2.34 | 2.32 | --- | 90 |
| 3 | 3.10 | 2.67 | 667 | --- |
| 7 | 2.33 | 2.76 | --- | --- |
| 8 | 1.26 | 1.80 | 615 | 109 |
| 9 | 2.07 | 6.29 | 663 | --- |
| 12 | 1.77 | 3.04 | --- | 107 |
| 15 | 2.78 | 2.14 | --- | --- |
| 16 | 3.23 | 8.76 | 797 | 124 |
| 20 | 2.02 | 3.28 | --- | --- |
| 23 | 2.03 | 3.29 | --- | --- |
| 33 | 2.44 | 2.53 | --- | 106 |
| 34 | 1.41 | 1.35 | --- | 113 |
| 35 | 2.69 | 2.75 | --- | --- |
| 36 | 4.34 | 10.30 | 617 | --- |
| 40 | 1.63 | 2.02 | --- | 110 |
| 46 | 2.40 | 5.21 | --- | 101 |
| 57 | 3.76 | 9.01 | 643 | --- |
| 59 | 4.46 | 8.27 | --- | --- |
| 74 | 1.83 | 2.32 | --- | 97 |
| 75 | 2.51 | 7.59 | --- | 89 |
| 76 | 2.06 | 4.83 | --- | 99 |
| 77 | 2.14 | 1.49 | --- | 91 |
| 78 | 2.39 | 5.94 | 687 | --- |
| 83 | 2.37 | 0.96 | --- | --- |
| 87 | 2.85 | 6.98 | 783 | 96 |
| 95 | 2.62 | 6.17 | 812 | --- |

**Table 6. Rat UMR-106 Bone Osteosarcoma $IC_{50}$ Values X $10^{-6}$M**

| Compound | Trypsin | Neutral Cathepsin | Elastase |
|---|---|---|---|
| 1 | 7.74 | 4.67 | 9.26 |
| 2 | 9.89 | 5.82 | 8.94 |
| 8 | 6.56 | 6.59 | 9.58 |
| 12 | 5.67 | 5.81 | 8.86 |
| 15 | 6.57 | 6.10 | 9.74 |
| 20 | 9.18 | 6.62 | 10.34 |
| 33 | 7.54 | 6.24 | 9.04 |
| 34 | 2.62 | 5.46 | 8.99 |
| 35 | 7.28 | 6.31 | 9.19 |
| 40 | 9.48 | 6.54 | 9.96 |
| 74 | 10.51 | 6.55 | 10.02 |
| 83 | 9.89 | 5.41 | 8.09 |

**Table 7. The Effects of Amine-carboxyboranes on Prostaglandin Cyclooxygenase and 5'-Lipoxygenase Activities $IC_{50}$ Values X $10^{-6}$ M**

| | Prostaglandin Cyclooxygenase | | | 5'-lipoxygenase | |
|---|---|---|---|---|---|
| Compound # | Macrophages | Be Sal Osteofbro | Beef Seminal Vesicle | Mouse Macrophage | Human Leukocytes |
| 1 | 4.19 | 3.94 | --- | --- | --- |
| 2 | 3.50 | --- | --- | 7.48 | 1.15 |
| 3 | 2.91 | --- | 3.26 | --- | 5.83 |
| 7 | --- | --- | 1.85 | --- | --- |
| 8 | 2.55 | 6.77 | 2.43 | 8.76 | 1.53 |
| 9 | 2.51 | --- | 1.92 | --- | --- |
| 12 | 3.53 | 5.49 | 7.60 | 9.80 | 4.77 |
| 15 | 2.81 | 4.24 | 2.86 | 6.61 | 5.37 |
| 16 | 2.41 | --- | 2.98 | --- | 5.37 |
| 23 | 5.19 | 8.05 | --- | 6.59 | 2.80 |
| 33 | 5.92 | 5.58 | --- | 9.89 | 3.47 |
| 34 | 5.88 | 4.26 | 5.90 | 8.07 | 6.43 |
| 36 | --- | --- | 2.23 | 6.67 | 5.67 |
| 40 | 3.05 | 9.29 | --- | 8.80 | 3.28 |
| 46 | 6.59 | 2.33 | 0.71 | --- | 6.39 |
| 57 | --- | --- | 3.09 | --- | --- |
| 74 | --- | --- | 1.52 | 6.31 | 4.06 |
| 75 | 6.61 | 3.68 | 4.31 | --- | --- |
| 76 | 1.61 | --- | 1.30 | --- | --- |
| 77 | 7.72 | 1.09 | 1.39 | --- | --- |
| 78 | 1.47 | 3.41 | 2.37 | --- | --- |
| 95 | --- | --- | 2.20 | --- | --- |

### REFERENCES

1. I.H. Hall, C.O. Starnes, Jr., B.F. Spielvogel, P. Wisian-Neilson, M.K. Das, and L. Wojnowick. *J. Pharm. Sci.* **68**, 685, (1979)
2. A. Sood, C.K. Sood, B.F. Spielvogel, and I.H. Hall. Eur. J. Med. Chem. 25, 301, (1990)
3. M.K. Das, P.K. Maiti, S. Roy, M. Mittakanti, K.W. Morse, and I.H. Hall. *Arch. Pharm.* **325**, 267, (1992)
4. K.G. Rajendran, B.S. Burnham, S.Y. Chen, A. Sood, B.F. Spielvogel, B.R. Shaw, I.H. Hall. *J. Pharm. Sci.*, in press.
5. B.F. Spielvogel, "Boron Chemistry 4," p. 119-129, R.W. Parry and G. Kodama Eds. *Pergamon Press New York*, 1980.
6. A. Sood, C.K. Sood, B.F. Spielvogel, I.H. Hall, O.T. Wong, M. Mittakanti, and K.W. Morse. *Arch. Pharm.* **324**, 423, (1991)
7. B.F. Spielvogel, F. Harchelroad, Jr., and P. Wisian-Neilson. *J. Inorg. Nucl. Chem.* **41**, 1223, (1979)
8. I.H. Hall, B.F. Spielvogel, A. Sood, F.U. Almed, and S. Jafri. *J. Pharm. Sci.* **76**, 359, (1987)
9. M. Mittankanti and K.W. Morse. *Inorg. Chem.* **29**, 554, (1990)
10. M.K. Das, P.K. Maitti, S. Roy, M. Mittakanti, K.W. Morse, and I.H. Hall, *Arch. Pharm.* **325**, 267, 1992.
11. B.F. Spielvogel, F.U. Ahmed, and A.T. McPhail. *Synthesis* **833**, (1986)
12. D.A. Ferkes, M.R.M.D. Charandabi, R. Livengood, K.W. Morse. Boron USA II, Reserach Triangle Park, NC 1990, p. 40.
13. B.F. Spielvogel, F.U. Ahmed, K.W. Morse, and A.T. McPhail. *Inorg. Chem.* **23**, 1776, (1984)
14. A. Sood and B.F. Spielvogel. *Main Group Metal Chem.* **12**, 143, (1989)
15. A.T. McPhail, K.D. Onan, B.F. Spielvogel, P. Wisian-Neilson. *J. Chem. Res.* (5) **205**, m 2601, (1978)
16. B.F. Spielvogel, A. Sood, K.W. Morse, D.T. Wong, and I.H. Hall. *Die Pharmazie* **46**, 592, (1991)
17. I.H. Hall, M.K. Das, F. Harchelroad, Jr., P. Wisian-Neilson, A.T. McPhail, and B.F. Spielvogel. *J. Pharm. Sci.* **70**, 339, (1981)
18. J.L. Peters, V.M. Norwood, III, and K.W. Morse. *Inorg. Chem.* **24**, 3713, (1986)
19. M.K. Das and S. Roy. *Synth. Reactions Inorg. Chem.* **15**, 53, (1985)
20. A. Sood, C.K. Sood, I.H. Hall, B.F. Spielvogel. *Tetrahedron* **47**, 6915, (1991)
21. M.P. Kaushik, M.R.M.D. Charandabi, M.L. Ettel, T.J. Lofthouse, and K.W. Morse. *Inorg. Chem.* **28**, 897, (1989)
22. M. Mittankanti, MRMD Charandabi, and K.W. Morse. *Inorg. Chem.* **29**, 3218, (1990)
23. Morse, et. al. to be filled in later.
24. C.K. Sood, A. Sood, B.F. Spielvogel, J.A. Yousef, B. Burnham, and I.H. Hall. *J. Pharm. Sci.* **80**, 1133, (1991)
25. P. Roszkowki, W.H. Rooks, A.J. Tomolonis, and L.M. Miller. *J. Pharmacol. Exp.* Ther. 179, 114, (1971)
26. C.A. Winter, E.A. Risley, and G.W. Nuss. *Proc. Soc. Exp. Biol. Med.* **111**, 544, (1962) L.C. Hendershot and J. Forsaith. *J. Pharmacol. Exp. Ther.* **125**, 237, (1959)
27. I.H. Hall, J.E. Hull, M. Mohseni, and F. Sajadi. *J. Pharm. Sci.* **69**, 1451, (1980)
28. L.F. Sancilio. *Proc. Soc. Exp. Biol. and Med.* **127**, 597, (1968)
29. B.H. Waksman, C.M. Pearson, and J.T. Sharp. *J. Immunol.* **85**, 403, (1960)
30. P.S. Chen, T.Y. Toribara, and H. Warner. *Anal. Chem.* **28**, 1257, (1956)
31. A.B. Roy. *Biochem. J.* **53**, 12, (1953)
32. I.H. Hall and G.L. Carlson. *J. Med. Chem.* **19**, 1257, (1976)
33. W.D. Schleuning and H. Fritz. *Methods Enzymol XLV,* 330, (1976)

34. J. Kleinerman, V. Ranga, J. Rynbrant, J.Sorenson, and J. Powers. *Am. Rev. Respir. Dis.* **121**, 381, (1980)
35. C.L. Hu, G. Crombie, and C. Franzblau. *Anal. Biochem.* **88**, 638, (1978)
36. T.E. Cawston and A.J. Barrett. *Anal. Biochem.* **99**, 340, (1979)
37. R.V.. Tomlinson, R.V. Ringold, M.C. Qureshi, and E. Forchielli. *Biochem. Biophy. Res. Comm.* **46**, 552, (1972)
38. M. Glatt, H. Kalin, K. Wagner, and K. Brune. *Agents and Action* **7**, 321, (1977)
39. D.L. Flynn, T.R. Belliotti, A.M. Boctor, D.T. Connor, C.R. Kostlan, D.E. Nies, D.F. Ortwine, D.J. Schrier, and J.C. Sircar. *J. Med. Chem.* **34**, 518, (1991).
40. D.L. Flynn, T. Capiris, W.J. Cetenko, D.T. Connor, R.D. Dyer, C.R. Kostlan, D.E. Nies, D.J. Schier, and J.C. Sirar. *J. Med. Chem.* **33**, 2070, (1990)
41. I.H. Hall, R. Simlot, C.B. Oswald, A.R.K. Murthy, H. ElSourady, and J.M. Chapman, Jr. *Acta. Pharm. Nord.* **2**, 387, (1990)
42. I.H. Hall, K.G. Rajendran, S.Y. Chem, O.T. Wong, A. Sood, and B.F. Spielvogel. *Agents and Action,* submitted.

**Received: March 22, 1994 - Accepted: April 19, 1994 - Received in camera-ready format: August 1, 1994**

